# Supplementary material for: Isolation and Identification of Chemical Compounds from Agaricus blazei Murrill and Their In Vitro Antifungal Activities
Source: Molecules. 2023 Oct 28;28(21):7321. doi: 10.3390/molecules28217321 (PMC10648600; doi:10.3390/molecules28217321)
Supplement: Supplementary file 1 [file molecules-28-07321-s001.zip › molecules-2606521-supplementary.pdf]

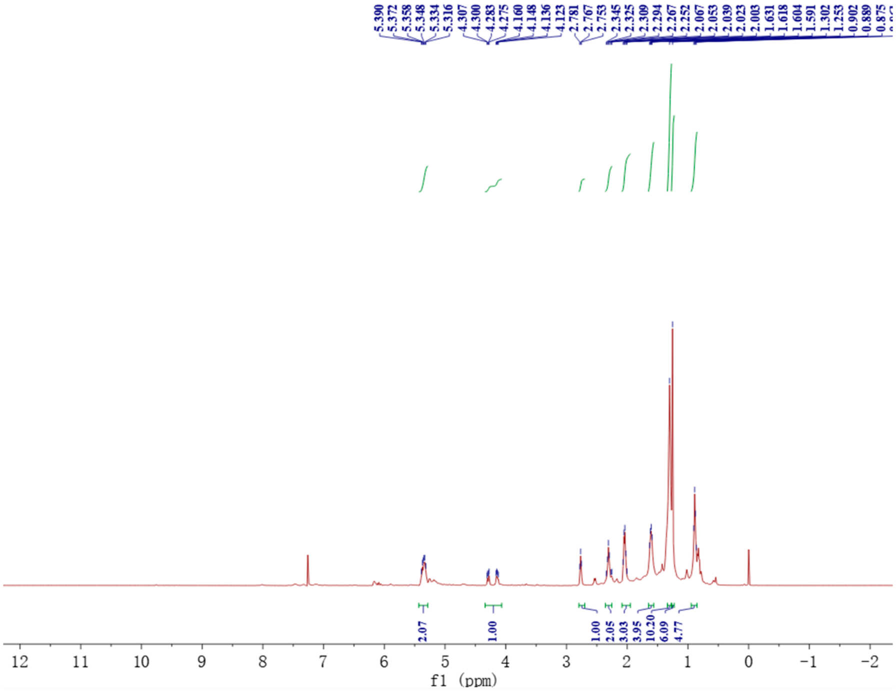

Figure S1, ^{1}H NMR spectrum of compound **1** in Methanod-*d₄*.

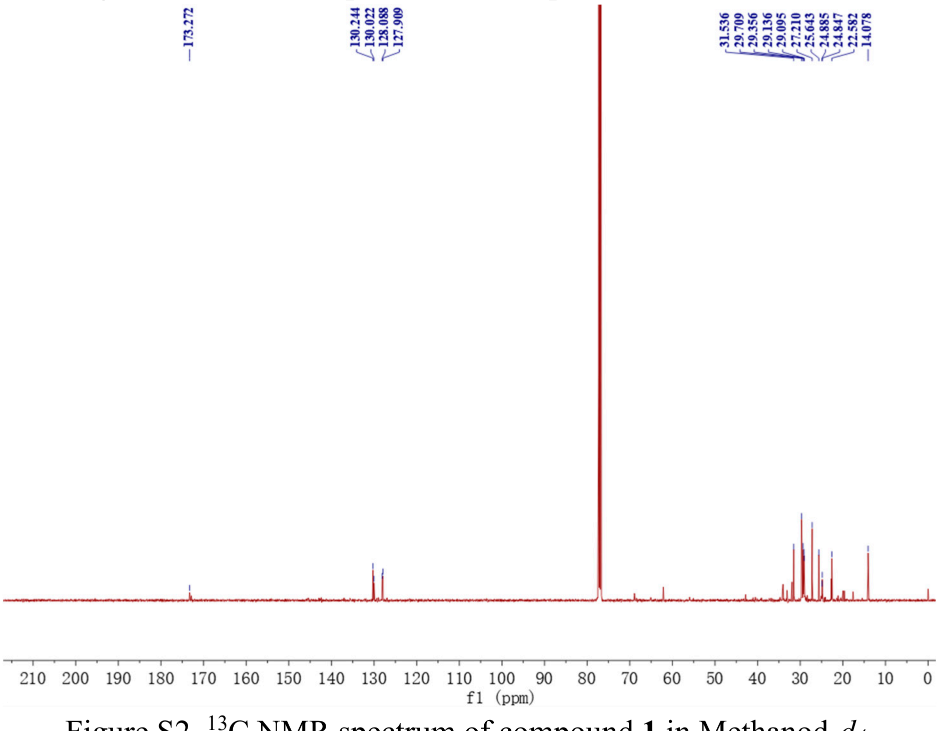

Figure S2, ^{13}C NMR spectrum of compound **1** in Methanod-*d₄*.

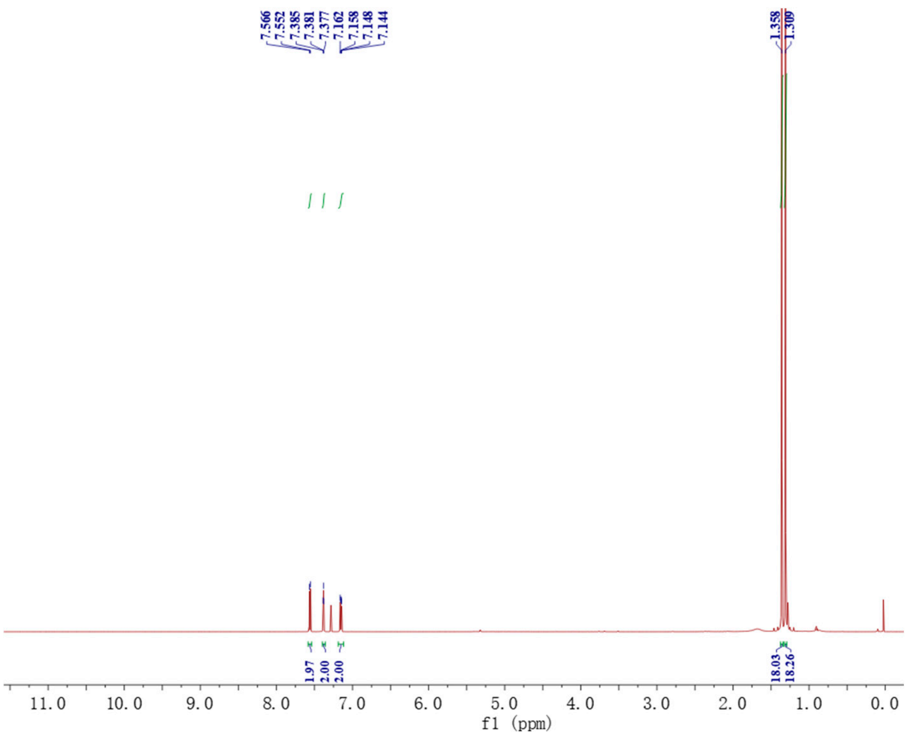

Figure S3, ^{1}H NMR spectrum of compound **2** in $CDCl_3$.

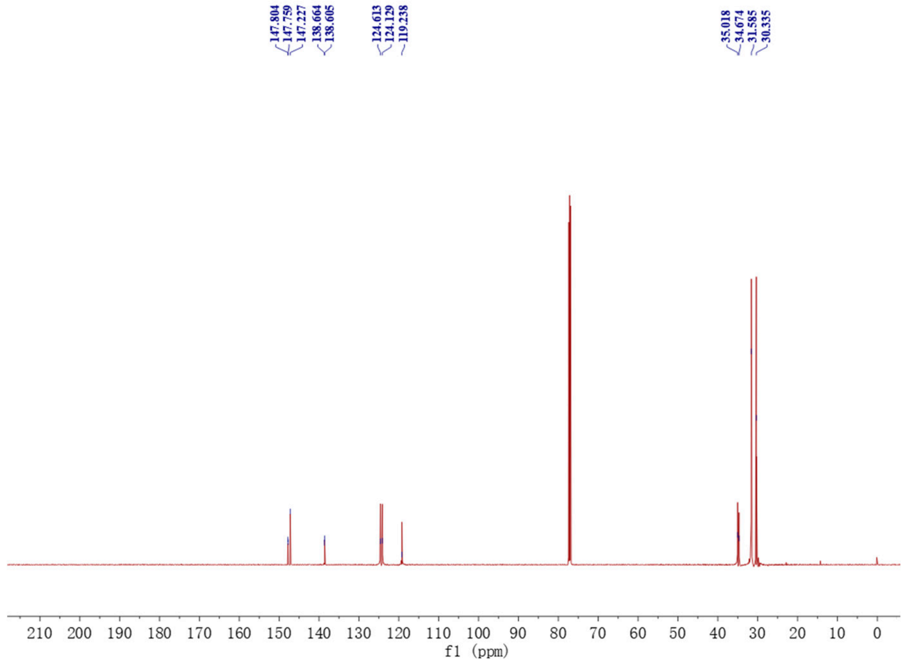

Figure S4, ^{13}C NMR spectrum of compound **2** in $CDCl_3$.

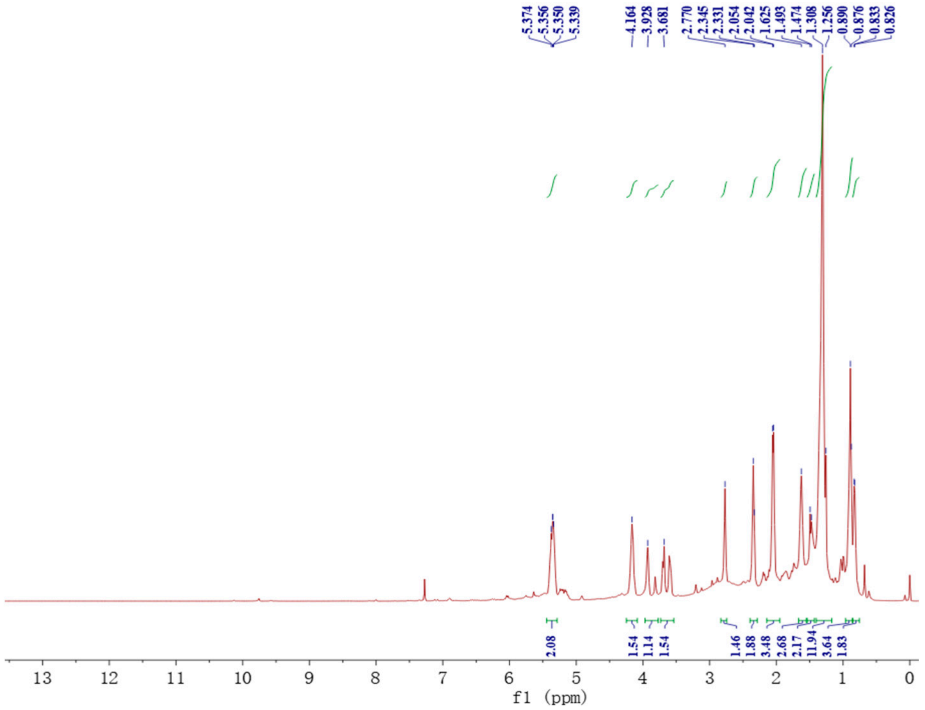

Figure S5, ^{1}H NMR spectrum of compound **3** in $CDCl_3$.

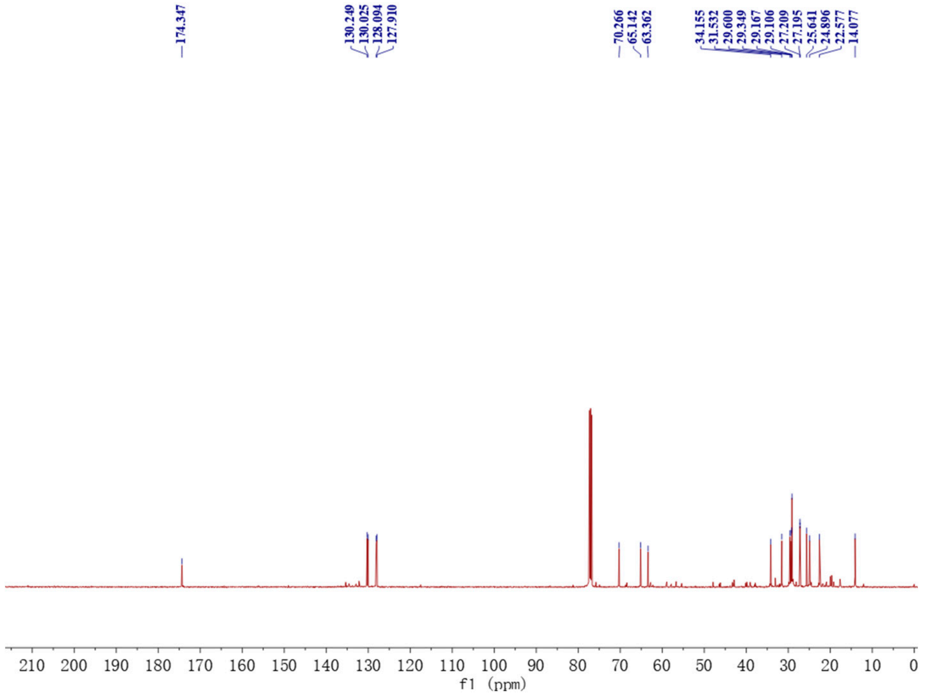

Figure S6, ^{13}C NMR spectrum of compound **3** in $CDCl_3$。

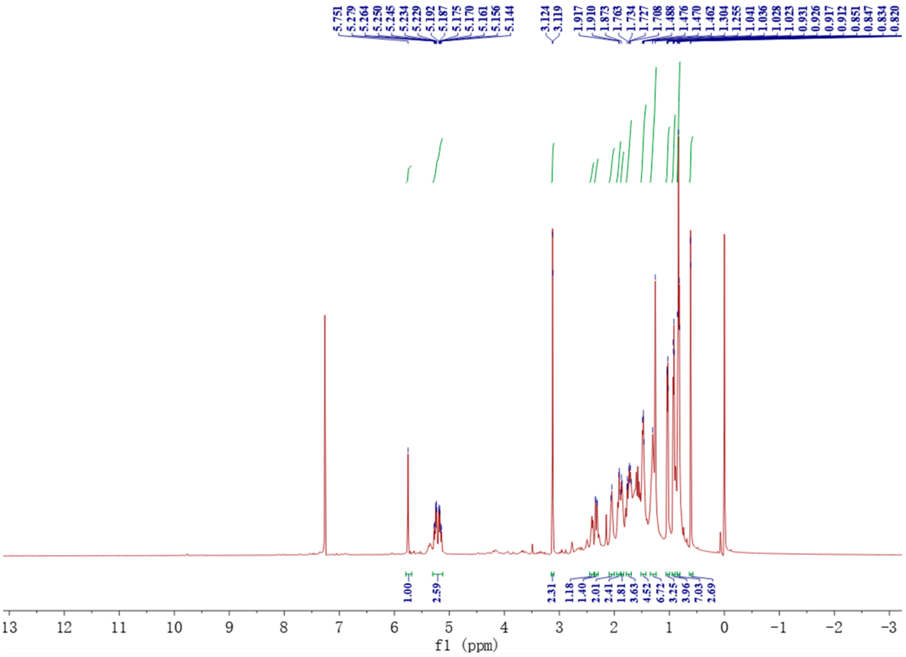

Figure S7, ^{1}H NMR spectrum of compound **4** in CDCl3

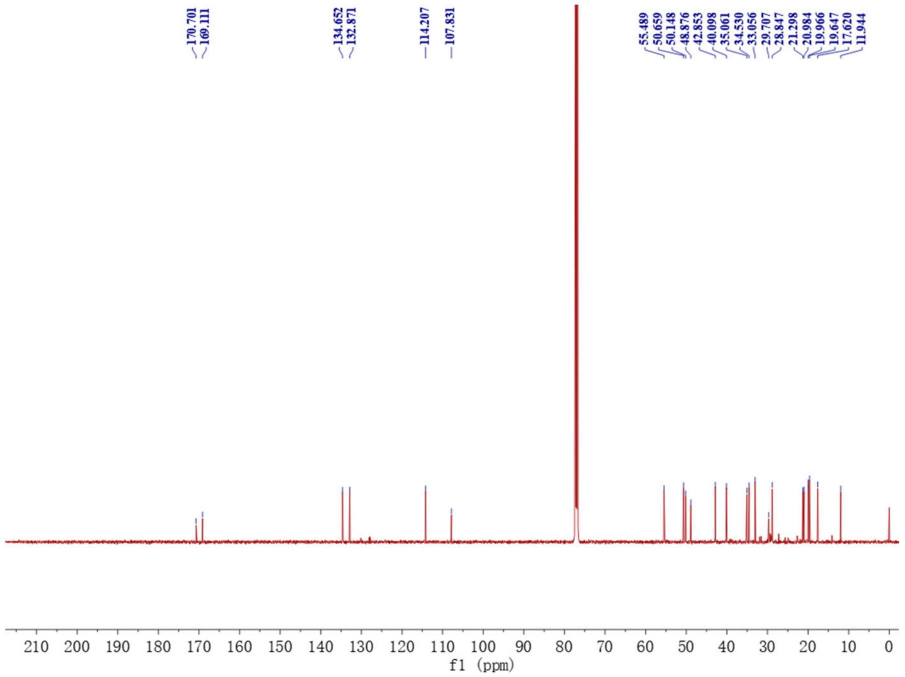

Figure S8, ^{13}C NMR spectrum of compound **4** in CDCl3.

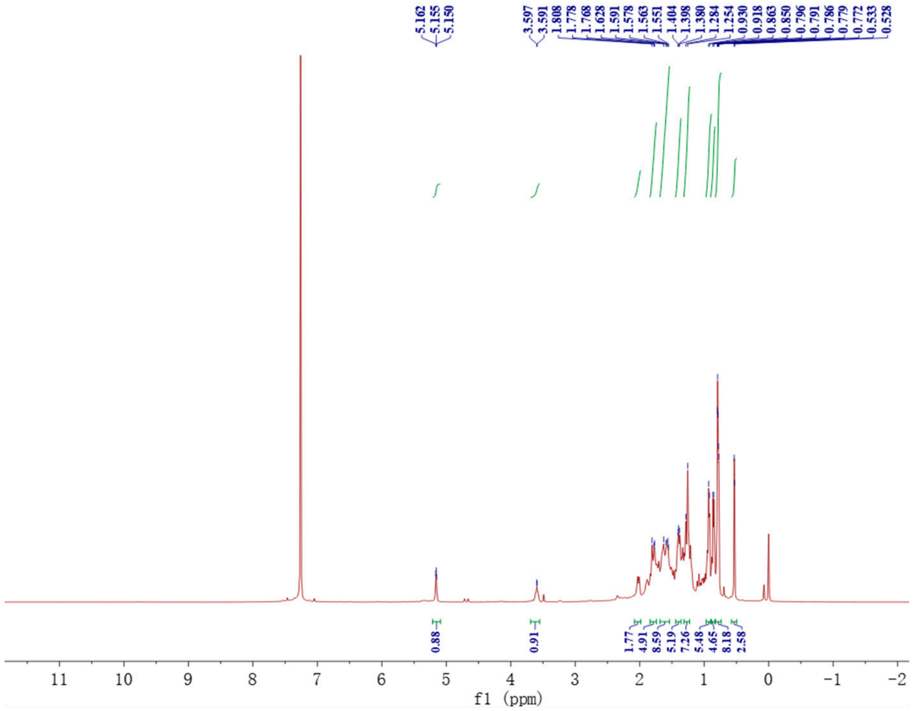

Figure S9, ^{1}H NMR spectrum of compound **5** in $CDCl_3$.

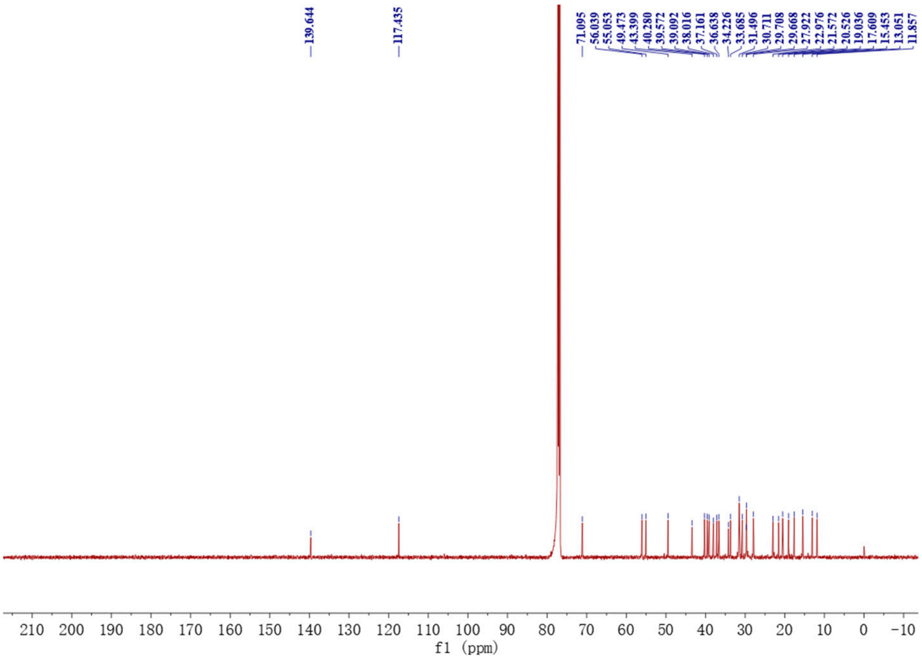

Figure S10, ^{13}C NMR spectrum of compound **5** in $CDCl_3$.

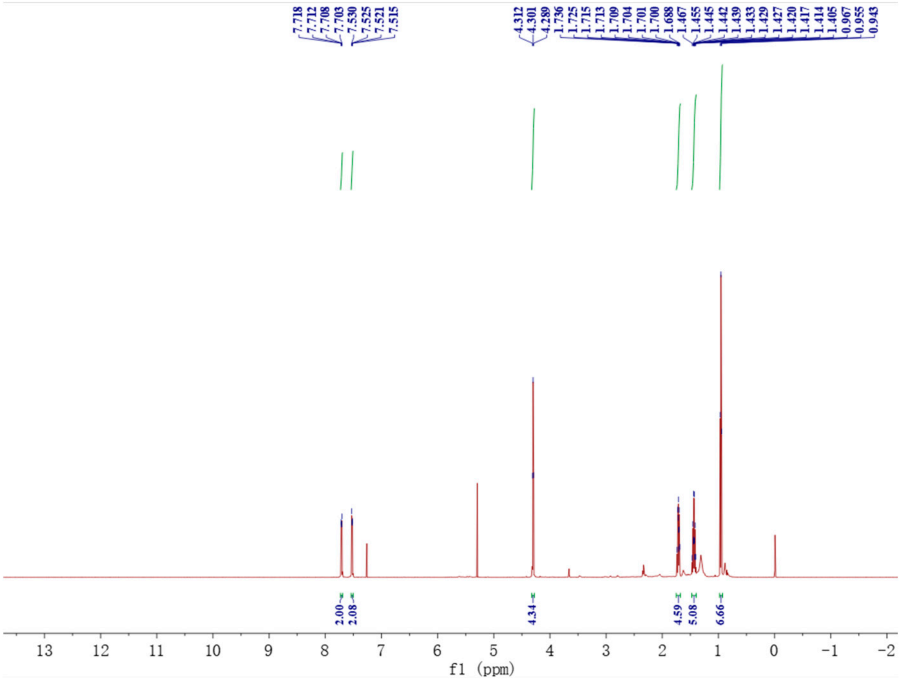

Figure S11, ^{1}H NMR spectrum of compound **6** in $CDCl_3$.

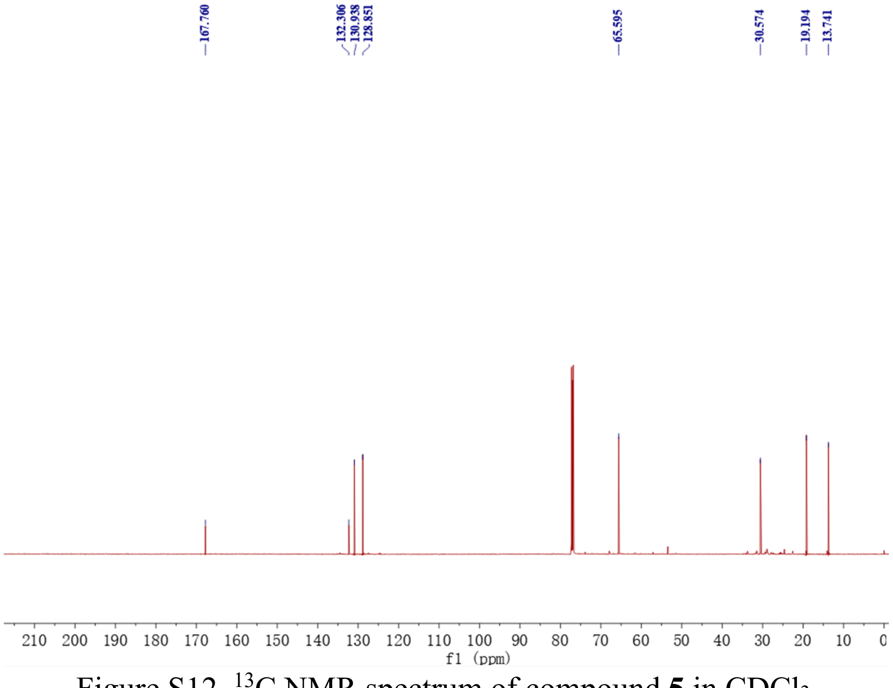

Figure S12, ^{13}C NMR spectrum of compound **5** in $CDCl_3$.
